# Emotional dysfunction in avoidant personality disorder and borderline personality disorder: A cross‐sectional comparative study

**DOI:** 10.1111/sjop.12771

**Published:** 2021-09-15

**Authors:** CHRISTINA FREDERIKSEN, OLE ANDRÉ SOLBAKKEN, RASMUS W. LICHT, CARSTEN RENÉ JØRGENSEN, MARIA RODRIGO‐DOMINGO, GRY KJAERSDAM TELLÉUS

**Affiliations:** ^1^ Psychiatric Clinic North Brønderslev Psychiatric Hospital Brønderslev Denmark; ^2^ Department of Clinical Medicine Aalborg University Aalborg Denmark; ^3^ Department of Psychology University of Oslo Oslo Norway; ^4^ Psychiatry Aalborg University Hospital Aalborg Denmark; ^5^ Department of Psychology Aarhus University Aarhus Denmark; ^6^ Psychology Department of Communication and Psychology Aalborg University Aalborg Denmark

**Keywords:** Affect integration, avoidant personality disorder, borderline personality disorder, affect integration inventory, emotional dysfunction

## Abstract

According to the literature, avoidant personality disorder (APD) is often overlooked in research on personality disorders. In the present study, patients with APD were compared to patients with borderline personality disorder (BPD) with respect to emotional dysfunction. Emotional dysfunction was operationalized through the Affect Integration Inventory. Sixty‐one patients receiving treatment at specialized outpatient hospital facilities for either BPD (*n* = 25) or APD (*n* = 36) (Diagnostic and Statistical Manual of Mental Disorders, fifth edition) were included in a cross‐sectional study. Supporting our expectations of no difference in the global capacity for affect integration between groups, the estimated difference was 0.00 (95% confidence interval [CI] [−0.53, 0.53]). On the other hand, the expected increased dysfunction in APD regarding Expression could not be confirmed. Furthermore, problems with specific affects distinguished the groups; integration of Interest was worse in APD (*p* = 0.01), whereas integration of Jealousy was worse in BPD (*p* = 0.04). In terms of prototypical modes of experiencing affects, APD was characterized by decreased access to the motivational properties of Interest (*p* < 0.01), while BPD was more driven by Interest (*p* < 0.01), Anger (*p* < 0.01), and Jealousy (*p* = 0.01). In conclusion, even though the two disorders are characterized by similar overall levels of emotional dysfunction, they differ systematically and predictably regarding specific affects and modes of experiencing. These findings carry implications for the understanding of emotional dysfunction in APD and BPD, suggesting specific areas of emotional dysfunction that could be targeted in tailored psychotherapeutic interventions.

## INTRODUCTION

Emotional dysfunction is inevitably associated with personality disorder (PD; American Psychiatric Association, 2013), and understanding the nature of such difficulties is crucial in treatment. While much research has been conducted on emotional dysfunctions in borderline personality disorder (BPD; Chapman, 2019; Daros & Williams, 2019), avoidant personality disorder (APD) appears relatively overlooked in the field (Lampe & Malhi, 2018; Wilberg, Karterud, Pedersen & Urnes, 2009). Accordingly, the aim of this study was to examine emotional dysfunction in patients with APD and compare them to emotional dysfunctions in patients with BPD. Emotional dysfunction was operationalized through the affect integration (AI) construct, defined as the functional integration of affects in cognition, motivation, and behavior (Solbakken, Hansen & Monsen, 2011). AI encompasses both the capacity to access and utilize the adaptive properties of discrete affects and a general capacity for emotion regulation. High levels of AI are thought to protect the individual against developing psychopathology (Monsen & Monsen, 1999; Solbakken, Hansen & Monsen, 2011).

According to the AI model, discrete affects can be defined as biologically founded and evolutionarily based responses that become idiosyncratically structured in each of us based on our unique developmental histories. In this process, our emotional life gradually organizes and becomes automatized in patterns or scripts for experiencing, comprehending, and expressing one’s affective reactions (Solbakken, Hansen & Monsen, 2011; Tomkins, 2008a, 2008b).

Affect integration is commonly operationalized by the observer‐rated Affect Consciousness Interview (ACI) but can also be operationalized through the self‐rated Affect Integration Inventory (AII). Both instruments aim to assess the level of adaptiveness of the individual’s affect organization. Various studies have demonstrated the validity of both the ACI (Lech, Andersson & Holmqvist, 2008; Monsen, Melgård & Ødegård, 1996; Solbakken, Hansen, Havik & Monsen, 2011; Taarvig, Solbakken, Grova & Monsen, 2015) and the AII (Frederiksen *et al*., 2021a; Solbakken & Monsen, 2021; Solbakken, Rauk, Solem, Lødrup & Monse, 2017).

Of particular relevance to the present study, the characteristics of emotional dysfunction in APD have previously been examined in a study by Johansen, Normann‐Eide, Normann‐Eide and Wilberg (2013) using the ACI. However, in terms of theoretical differentiation and as empirically demonstrated in a recent study by Frederiksen *et al*. (2021a), the AII taps into components and dimensions of the AI construct other than the ACI. Thus, use of the AII may lead to new insights into emotional dysfunction related to APD.

### Emotional dysfunction in APD

In the literature, APD has been linked to dysfunctional regulation and maladaptive management of emotions. Millon (1981) asserts that individuals with APD protect themselves from real and imagined psychological pain by breaking up, destroying, or repressing painful thoughts and emotions. Beck and Freeman (1990) view individuals with APD as characterized by a heightened sensitivity towards feelings of sadness and anxiety, hyperawareness of painful feelings, and cognitive avoidance of identifying unpleasant thoughts.

While some researchers have suggested that individuals with APD keep thoughts and emotions away from their consciousness for defensive reasons, others have stressed that the lack of emotional clarity is primarily due to deficits in their capacity to access thoughts and feelings (Dimaggio *et al*., 2007). Procacci, Popolo, Petrilli and Dimaggio (2007) proposed that individuals with APD mainly experience metacognitive malfunctioning in identifying thoughts and emotions and in understanding the reasons behind their reactions. On similar lines, Jordet and Ladegård (2018) theorize that the lack of access to emotional reactions is at the center of emotional dysfunction in APD. They suggest that dysfunction in affective causality leads individuals with APD to not know how or why they are feeling the way they do. Nørgaard and Simonsen (2019) addressed the issue of affect regulation in cluster C PDs by conceptualizing the pattern as fluctuating between lack of access and being overwhelmed by emotions. It has been suggested that individuals with APD try to avoid emotional reactions and tend to lose control when failing, leading to a dysregulated reaction coupled with anxiety (Nørgaard & Simonsen, 2019).

Despite ample theorizing, only a small number of empirical studies have examined emotional dysfunction in individuals with APD. Taylor, Laposa and Alden (2004) examined emotional avoidance and found that individuals with APD exhibited greater avoidance of both positive and negative emotions. In a student sample, Ye, Yao, Wen‐Qing and Kong (2011) observed a greater degree of negative and a lesser degree of positive emotions in individuals with APD (selected according to the Personality Diagnostic Questionnaire and Personality Disorder Interview‐IV) compared to healthy controls (Ye *et al*., 2011). Spinhoven, Bamelis, Molendijk, Haringsma and Arntz (2009) found that patients with cluster C PDs reported significantly more experiential avoidance, defined as an unwillingness to be in contact with or a tendency to alter the form or frequency of particular unwanted private experiences, compared to nonclinical controls (Spinhoven *et al*., 2009).

Several studies have linked cluster C PDs and APD in particular to high rates of alexithymia (Bach, de Zwann, Ackard, Nutzinger & Mitchell, 1994; Joyce, Fujiwara, Cristall, Ruddy & Ogrodniczuk, 2013; Nicolo *et al*., 2011), which is defined as deficient processing of emotional experiences leading to difficulties with identifying and labeling emotions (Bagby, Parker & Taylor, 1994). However, a study by Simonsen *et al*. (2020) demonstrated large variation in the distribution of alexithymia (measured by TAS 20) in patients with APD. Moroni *et al*. (2016) examined the meta‐cognitive profile for APD compared to other PDs and identified dysfunctions in the ability to identify their own inner state and to correctly identify the mental state of others as distinct features of APD (Moroni *et al*., 2016).

Regarding management of discrete emotions, a study by Schoenleber and Berenbaum (2010) conducted in a sample of students found that individuals with cluster C traits tended to describe shame as an especially unpleasant experience and as an aversive emotion. Furthermore, these individuals also seemed more prone to experience shame reactions. More specifically, Schoenleber and Berenbaum (2010) found that individuals highly prone to experience shame only displayed greater levels of cluster C symptoms when this shame proneness was combined with high level shame aversion (Schoenleber & Berenbaum, 2010). In a later study, Schoenleber and Berenbaum (2012) reported that shame aversion was related to several PD symptoms, while shame proneness was only related to APD symptoms (Schoenleber & Berenbaum, 2012).

Finally, in a study by Karterud *et al*. (2016), the relationship between primary emotions and PDs was examined. In this context, primary emotions were defined according to Panksepp (2005) as cross‐species emotional systems that work as prime motivators that include behavior and autonomic response patterns, as well as primal affective feeling states (Karterud *et al*., 2016; Panksepp, 2005; Panksepp & Watt, 2011). Results from the study indicated that compared to other PDs, APD was characterized by a low threshold for fear (i.e., the affective reaction is easy to evoke) and a heightened threshold for Play and Seeking (i.e., the affective reaction is difficult to evoke). Furthermore, an increased level of anger was related to a reduced number of APD criteria (Karterud *et al*., 2016).

### Emotional dysfunction in BPD

Suicide attempts, nonsuicidal self‐injury, and self‐damaging impulsive behaviors (e.g., substance abuse or risky sexual behavior) are some of the well‐known manifestations of BPD (Chapman, 2019; Links, Gould & Ratnayake, 2003; Rosenthal, Cukrowicz, Cheavnes & Lynch, 2006; Sansone & Sansone, 2011). Being a hazard to life, these behaviors might be interpreted as expressions of emotional dysfunction that serve as coping strategies while tending to fail miserably in reducing distress in the long term (Carpenter & Trull, 2013).

According to Linehan’s biosocial model (1993), emotional dysfunction in BPD consists of four components: emotional sensitivity, heightened negative affect, deficient emotional regulation strategies, and frequent use of maladaptive regulation strategies (Linehan, 1993). Being by far the most studied of all PDs, a substantial amount of evidence supports the notion of emotional dysfunction as underlying BPD, a pattern characterized by heightened emotional sensitivity and hyperreactivity towards emotional stimuli (Berking & Wupperman, 2012).

A review by Chapman (2019) summarized the manifestations of emotional dysfunction in BPD, asserting that individuals with BPD often experience difficulties with emotional clarity and awareness, identification, and description of emotions. Additionally, a tendency to be less specific in the differentiation of various negative emotions, consequently representing all of them as the same, was further demonstrated. The lack of clarity regarding emotional states might hinder the use of efficient emotional regulation strategies, with studies suggesting that BPD is indeed correlated with restricted access to such strategies, favoring short‐term and ineffective strategies (Chapman, 2019; Daros & Williams, 2019). In a meta‐review, Daros and Williams (2019) found evidence that BPD was associated with increased use of ineffective emotional regulation strategies, such as rumination and avoidance, and reduced use of more adaptive strategies, such as problem solving and acceptance, when compared to other mental disorders, for example, social anxiety and bipolar disorder (Daros & Williams, 2019). Finally, evidence supports the idea that suppression is significantly associated with BPD and that individuals with BPD report fewer emotional benefits from acceptance than from suppression (Baer, Peters, Eisenlohr‐Moul, Geiger & Sauer, 2012; Chapman, Rosenthal, Dixon‐Gordon, Turner & Kuppens, 2017).

### Emotional dysfunction in APD compared to BPD

Few studies have directly compared APD and BPD with respect to the severity and distinctness of the emotional dysfunction characterizing each of the PDs. In a study by Herpertz *et al*. (2000), the emotional responses in APD and BPD were compared, and data did not support the hypotheses of general affective hypersensitivity in BPD (Herpertz *et al*., 2000). Applying the ACI, Johansen *et al*. (2013) compared emotional dysfunction between APD and BPD. Results suggested that the global capacity to adaptively perceive, tolerate and comprehend affective experiences was different in the two groups, with the APD group scoring significantly lower. Additionally, the APD group proved more impaired in their ability to communicate their own affective experiences in a direct and clear manner. Finally, the two groups differed in their capacity for AI in the discrete affects interest and contempt, with the APD group scoring significantly lower than the BPD group (Johansen *et al*., 2013).

To summarize, emotional difficulties in APD might be considered a comprehensive defensive strategy where individuals with APD have the capacity to adequately identify feelings but keep them away from consciousness. Alternatively, emotional dysfunction can be considered a malfunction in the capacity to correctly identify emotions and their cause. However, as noted by Dimaggio *et al*. (2007), one position does not necessarily exclude the other, since individuals with APD also succeed in cognitive avoidance (a defense strategy) when they are unable to keep an emotional reaction in memory long enough to understand the reason behind it. In contrast, emotional dysfunction in BPD seems to be characterized by a lack of emotional awareness and clarity, along with a tendency to represent negative emotions in an undifferentiated manner. It appears that both APD and BPD manifest as dysfunctions in the capacity to perceive, tolerate, and comprehend emotional reactions. However, they diverge, as APD may be characterized by too little emotional information and activation, whereas BPD appears overwhelmed by it. Regarding discrete affects, the existing evidence indicates that individuals with APD experience increased dysfunction in the management of interest and fear and BPD in the management of anger, while dysfunctions in the management of shame appear to be of equal importance in both groups (Schoenleber & Berenbaum, 2010, 2012).

### The present study

In this study, we compared patients with APD to patients with BPD with respect to emotional dysfunction. We hypothesized that both APD and BPD are characterized by equivalent dysfunctions in the general capacity to perceive, tolerate, and comprehend discrete affects. Further, we expect the capacity to communicate and share affective reactions and experiences with others (measured as Expression on the AII) to differ between the two. Finally, we expect that emotional dysfunction in APD and BPD will differ in terms of characteristic dysfunctional modes of experiencing specific affects. Based on the diagnostic conceptualization of APD and BPD in the DSM‐5 (American Psychiatric Association, 2013), we expect BPD to be more *Driven by Interest, Driven by Anger and Driven by Jealousy,* while we expect APD to lack *Access to Interest* and *Access to Anger*.

## METHODS

### Study participants

Study participants were recruited among patients with a diagnosis of PD referred and treated at a specialized outpatient unit of Aalborg University Hospital or a specialized outpatient unit of Brønderslev Psychiatric Hospital from October 2015 to December 2018. Inclusion criteria were a primary diagnosis of either APD or BPD according to the Diagnostic and Statistical Manual of Mental Disorders (DSM‐5; American Psychiatric Association, 2013), age above 18 years, Danish literacy and obtaining written informed consent. Exclusion criteria were co‐occurring APD and BPD, a diagnosis of schizotypal PD or antisocial PD, comorbid psychotic disorder, bipolar I disorder, developmental disorder (e.g., Asperger’s disorder), or a primary diagnosis of drug/alcohol dependence (e.g., substance use interfering with ratings).

### Design, procedures and research ethics

Data used in this cross‐sectional study constitute part of an overarching study of various aspects of AI in PDs (Frederiksen *et al*., 2021a, 2021b).

Diagnostics were established after conducting Present State Examination (PSE; SCAN Advisory Committee, 2002) and the Structured Clinical Interview for DSM‐IV Axis II Personality Disorders (SCID‐II; First, 1994). Semi‐structured interviews were conducted by experienced psychiatrists and psychologists who received formal training beforehand and throughout the data collection process. Final diagnoses were determined according to the DSM‐5 (American Psychiatric Association, 2013).

Data on AI, symptom distress, interpersonal problems, and perceived quality of life were collected using self‐reported instruments through an online survey provider, SurveyXact.

All patients were informed that participation was voluntary, and that nonparticipation would not affect their treatment in any way. Written and oral information about the study was provided before ratings. This study was conducted in accordance with the Declaration of Helsinki and was approved by the Danish Data Protection Agency (2019‐017816). No further approval was needed from the Danish National Committee on Biomedical Research Ethics due to the nature of the study.

### Measures

#### Quality of life

Quality of life was assessed by the WHOQOL‐BREF (World Health Organization & Division of Mental Health, 1996). Low scores indicate poorer life quality. Cronbach’s alpha values for the sample were 0.72 for the physical health domain, 0.78 for the psychological health domain, 0.56 for the social relationship domain and 0.60 for the environment domain.

#### Symptom distress

The Symptom Checklist‐90, Revised (SCL‐90‐R; Derogatis, 1994), is a well‐established self‐reported scale to assess symptom distress and psychopathological symptoms. The global severity index (GSI) is calculated as an average score across all items, indicating the current level of distress. McDonald’s ω for the sample was 0.97 for GSI.

#### Interpersonal problems

The Inventory of Interpersonal Problems 64 circumplex version (IIP‐64; Horowitz, Alden, Wiggins & Pincus, 2000) was applied to determine the level of general interpersonal problems. McDonald’s ω for the sample was 0.92 for IIP‐Global.

#### Emotional dysfunction/AI

The level of AI was measured using the self‐reported instrument AII (Solbakken *et al*., 2017). The instrument consists of 112 statements about perceived awareness, tolerance, and expression of nine discrete affects, namely, Interest, Joy, Fear, Anger, Shame, Sadness, Jealousy, Guilt, and Tenderness. Eighty‐two items are indicators of experience capacity, and 30 items are indicators of expression capacity. Each item is rated on a ten‐point Likert scale ranging from does not fit at all (0) to fits perfectly (9). Higher scores correspond to increased levels of AI. Items are phrased so they tap into the level of adaptive experience or expression of the affects. AII scores can be divided into three levels: an overall mean score across all items (Global AI), a mean score for the 82 items tapping into the experience of affects (Experience), a mean score for the 30 items tapping into the expression of affects (Expression) and separate mean scores for the nine discrete affects (e.g., Anger, Joy). In addition, the AII contains additional scales for representing emotional dysfunction in terms of characteristic modes of experiencing affects, for example, whether one tends to experience too little or too much (Greenberg & Bolger, 2001). On the AII, these processes are defined as either the tendency to be driven by or to experience lack of access to, for example, Anger. High scores on the *Driven by* scales imply affective underregulation, carrying an increased risk of being overwhelmed by the affect, a tendency to lose control over the affective expression, to act out and to engage in impulsive behavior. Low scores on the *Access to* scales imply affective overregulation. An individual with low access to the adaptive properties of affects appears constricted and struggles with the understanding of the motivational aspect of discrete affects. Therefore, high scores on the *Driven by* scales and low scores on the *Access to* scales indicate problems with AI. McDonald’s ω for the AII scales in the current sample were 0.94 for Global AI, 0.90 for Experience, and 0.91 for Expression. Cronbach’s alpha values for the discrete affects in this sample were 0.68 for Sadness, 0.78 for Anger, 0.86 for Tenderness, 0.75 for Guilt, 0.80 for Fear, 0.77 for Shame, 0.82 for Interest, 0.80 for Joy, 0.91 for Jealousy, 0.64 for *Driven by Anger*, 0.76 for *Driven by Guilt*, 0.77 for *Driven by Shame*, 0.59 for *Access to Anger*, 0.80 for *Access to Guilt*, 0.78 for *Access to Interest*, 0.83 for *Access to Joy*, and 0.84 for *Access to Tenderness*.

### Statistical analyses

Summary statistics included the mean and standard deviation for continuous variables and the frequency and percentage in each category for categorical variables separately for the APD and BPD groups. The categorical variables were compared between groups using Fisher’s exact test, while the continuous variables were compared using two‐sided *t*‐tests assuming unequal variances. Differences between groups regarding the AII scores and the *Access to* and *Driven by* scales were analyzed using *t*‐tests assuming unequal variances for the two groups. For the variables where no difference between the groups was hypothesized, two‐tailed *p*‐values are reported. For those where the BPD score is hypothesized to be higher than the APD score, upper‐tail *p*‐values are reported instead. To appraise the between‐group differences, Cohen’s *d* was computed and interpreted according to convention (*d* = 0.20–0.50, small; *d* = 0.50–0.80, moderate; *d* > 0.80, large; Cohen, 1988). Analyses were performed in Stata 16 (StataCorp., 2019). Results with *p*‐values < 0.05 were considered statistically significant.

## RESULTS

### Characteristics of the patient groups

A total of 61 patients participated in the study; 36 were diagnosed with APD, and 25 were diagnosed with BPD. Demographic characteristics are shown in Table 1. The only characteristics that are statistically significantly different between the groups are sex (all females in BPD compared to 75% females in APD, *p*‐value = 0.02) and educational level, where three times as many had completed high school in the APD group compared to the BPD group (*p*‐value = 0.03). Self‐harm was more common in the BPD group (36%) than in the APD group (12%), though this difference is not statistically significant (*p*‐value = 0.05). Regarding IIP‐64, GSI, or perceived quality of life, the mean scores of each of these variables were very similar for both groups, except in the social relationship domain, where the APD group scored 1.2 points lower. Suicide attempts, substance abuse, eating disorders, and behavioral disorders were rare in both groups (below 8%).

**Table 1:** Demographic and clinical characteristics of the participants

	BPD (*n* = 25)	APD (*n* = 36)	*p*‐value
Female sex	25 (100%)	28 (78%)	0.02
Age (years)^a^	27.8 (6.9)	31.6 (8.7)	0.07
Married/cohabiting^b^	12 (48%)	21 (62%)	0.4
Completed high school^b^	4 (16%)	15 (44%)	0.03
Mood disorder	5 (20%)	11 (31%)	0.4
Anxiety disorder	5 (20%)	10 (28%)	0.6
Self‐harm^b^	9 (36%)	4 (12%)	0.05
No. of PD‐diagnoses	1.4 (0.5)	1.4 (0.56)	0.8
GSI	1.9 (0.6)	1.8 (0.6)	0.4
IIP‐64 global score^c^	1.7 (0.5)	2.0 (0.5)	0.08
WHOQOL‐BREF			
Quality of life perception	2.6 (1.0)	2.3 (0.8)	0.2
Health perception	2.5 (1.2)	2.3 (0.9)	0.5
Physical health domain	9.9 (1.9)	10.0 (2.0)	0.9
Psychological health domain	9.5 (2.7)	9.0 (1.6)	0.4
Social relationships domain	11.0 (3.5)	9.8 (2.9)	0.2
Environment domain	11.8 (2.5)	11.7 (1.9)	0.8

Note

ABP = avoidant personality disorder; BPD = borderline personality disorder; GSI = global severity index; PD = personality disorder.

^a^ Information missing for one APD patient.

^b^ Information missing for two APD patients.

^c^ Information missing from one APD and one BPD patient.

### AI scores in APD and BPD groups

Table 2 shows the mean and standard deviations for AII scores in the APD and BPD groups. At the overall level of emotional dysfunction, as represented by Global AI, the means and standard deviations were the same in both groups. Statistically significant differences between the groups were observed for the specific affects Interest (BPD > APD) and Jealousy (APD > BPD) with moderate effect sizes based on their Cohen’s *d*. Difference in Expression was not statistically significant.

**Table 2:** Scores for global‐AI, experience, expression and discrete affects in the BPD and APD groups (mean and standard deviation), estimated mean difference between groups with 95% CI, and Cohen’s d

	BPD (*n* = 25)	APD (*n* = 36)	Difference	*p*‐value	Cohen’s *d*
Global‐AI	3.7 (1.0)	3.7 (1.0)	−0.00 [−0.53, 0.53]	>0.9	−0.00 [−0.51, 0.51]
Experience	3.5 (1.0)	3.7 (1.0)	−0.21 [−0.74, 0.33]	0.4	−0.20 [−0.71, 0.31]
Expression	4.2 (1.4)	3.6 (1.4)	0.54 [−0.18, 1.25]	0.07	0.39 [−0.12, 0.91]
Interest	4.9 (1.4)	4.0 (1.3)	0.97 [0.29, 1.65]	0.01	0.75 [0.22, 1.27]
Jealousy	3.2 (2.2)	4.5 (2.4)	−1.23 [−2.43, −0.03]	0.04	−0.53 [−1.05, −0.01]
Guilt	4.1 (1.4)	4.8 (1.4)	−0.72 [−1.46, 0.03]	0.06	−0.50 [−1.02, 0.02]
Joy	3.9 (1.6)	3.3 (1.3)	0.63 [−0.13, 1.38]	0.1	0.43 [−0.09, 0.95]
Tenderness	4.8 (1.9)	4.4 (1.8)	0.36 [−0.59, 1.31]	0.5	0.20 [−0.32, 0.71]
Sadness	3.3 (1.3)	3.1 (1.2)	0.19 [−0.44, 0.83]	0.5	0.16 [−0.35, 0.67]
Fear	2.9 (1.4)	3.0 (1.3)	−0.12 [−0.82, 0.58]	0.7	−0.09 [−0.60, 0.42]
Shame	3.1 (1.5)	3.0 (1.3)	0.11 [−0.60, 0.82]	0.8	0.08 [−0.43, 0.59]
Anger	3.1 (1.2)	3.2 (1.6)	−0.10 [−0.86, 0.65]	0.8	−0.07 [−0.58, 0.44]

Note

All *p*‐values from two‐sided *t*‐tests except for Expression (upper tailed *p*‐value).

ABP = avoidant personality disorder; AI = affect integration; BPD = borderline personality disorder; CI = confidence interval.

### Differences in prototypical modes of experiencing affects

**Fig. 1:**
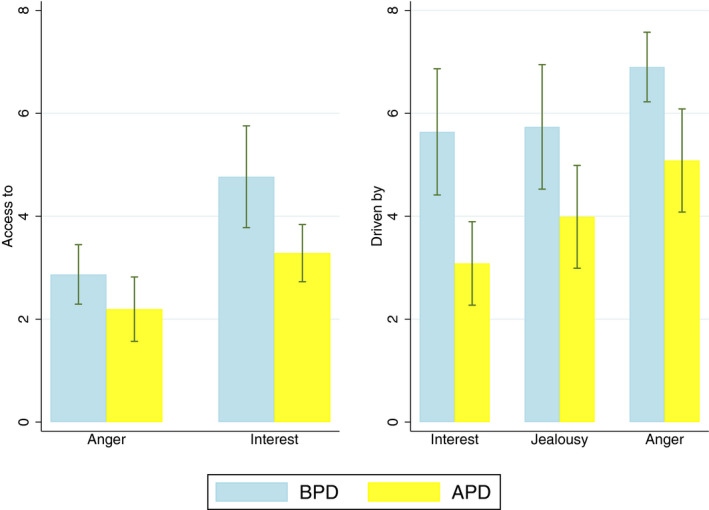
Barplots showing the mean and 95% CI for the *Access to* and *Driven by* scales hypothesized to be different between BPD and APD. For *Access to* lower scores indicate more problems, while for *Driven by* higher scores indicate more problems. ABP = avoidant personality disorder; BPD = borderline personality disorder; CI = confidence interval.

In Table 3, means and standard deviations for the scores in the *Access to* and *Driven by* scales are displayed, as well as the estimated mean differences between groups with 95% CIs. For most scales with hypothesized differences (see Fig. 1) between APD and BPD, the differences between groups were statistically significant with moderate to large effect sizes. The APD group had significantly lower *Access to Interest* than the BPD group. The difference in *Access to Anger* was not statistically significant, although the mean score was lower in the APD group. For the *Driven by* scales, including Jealousy, Anger, and Interest, the BPD group was significantly more driven than the APD group.

**Table 3:** Mean and standard deviation for the Access to and Driven by variables in each group, estimated mean difference (95% CI) between the groups, p‐value for the difference, plus Cohen’s d

	BPD (*n* = 25)	APD (*n* = 36)	Difference	*p*‐value	Cohen’s *d*
Access to					
Interest	4.8 (2.4)	3.3 (1.6)	1.48 [0.37, 2.60]	0.01^a^	0.75 [0.22, 1.27]
Anger	2.9 (1.4)	2.2 (1.8)	0.68 [−0.16, 1.51]	0.06^a^	0.40 [−0.12, 0.92]
Guilt	4.8 (2.4)	5.7 (2.0)	−0.90 [−2.06, 0.26]	0.1^b^	−0.42 [−0.94, 0.10]
Tenderness	4.7 (2.5)	5.1 (2.3)	−0.40 [−1.66, 0.87]	0.5^b^	−0.17 [−0.68, 0.34]
Joy	2.1 (2.0)	2.2 (1.5)	−0.09 [−1.05, 0.88]	0.8^b^	−0.05 [−0.56, 0.46]
Driven by					
Interest	5.6 (3.0)	3.1 (2.4)	2.56 [1.11, 4.00]	<0.01^a^	0.97 [0.42, 1.50]
Anger	6.9 (1.6)	5.1 (3.0)	1.82 [0.63, 3.00]	<0.01^a^	0.72 [0.19, 1.25]
Jealousy	5.7 (2.9)	4.0 (3.0)	1.75 [0.21, 3.28]	0.01^a^	0.59 [0.07, 1.11]
Guilt	7.4 (2.0)	6.4 (2.7)	1.00 [−0.19, 2.18]	0.1^b^	0.41 [−0.10, 0.93]
Shame	5.4 (2.5)	5.9 (2.1)	−0.52 [−1.75, 0.71]	0.4^b^	−0.23 [−0.74, 0.29]

Note

Within each subscale, the affects are sorted by ascending *p*‐value.

ABP = avoidant personality disorder; BPD = borderline personality disorder; CI = confidence interval.

^a^ Upper‐tailed *p*‐value from *t*‐test.

^b^ Two‐sided *p*‐value from *t*‐test.

## DISCUSSION

This study is the first to compare emotional dysfunction in APD and BPD using the AII. Sixty‐one patients with either APD or BPD were recruited from psychiatric outpatient clinics specializing in the treatment of PDs. It was expected that APD and BPD would be characterized by comparable levels of dysfunction in the overall capacity to perceive, tolerate, and understand affective experiences, while we expected APD to be more impaired in the capacity to express affective states in an adaptive manner. What would more clearly separate the groups, we hypothesized, would be the integration of selected discrete affects and prototypical modes of experiencing such affects. Based on theoretical conjectures and prior studies, we expected that APD would primarily lack access to the adaptive and motivational properties of affects, while BPD would more often become overwhelmed, lose control over, and be driven by affective reactions.

### Emotional dysfunction in patients with APD

When comparing patients with APD to patients with BPD, we observed highly similar levels of overall emotional dysfunction as measured by Global AI and Experience. A study by Wilberg *et al*. (2009) established that levels of psychosocial dysfunction in terms of functional impairment and subjective distress associated with APD are comparable to the levels detected in BPD. In this study, our focus was beyond symptoms and general functioning, and we report data suggesting that APD and BPD have comparable levels of impairment in the structural capacity for perceiving, tolerating and comprehending affective reactions (Frederiksen *et al*., 2021b). Regarding the capacity to adaptively communicate affective states, the two groups differed slightly more with a lower mean score in the APD group. However, the difference was smaller than expected and was not statistically significant. Hence, this result indicates that the level of dysfunction in the capacity to genuinely communicate one’s affective states is similar in patients with either APD or BPD. However, the implications for communication appear different, regardless of whether one has a pattern of emotional instability or emotional avoidance. Using the ACI in similar patient groups as the present study, Johansen *et al*. (2013) observed a statistically significant difference between groups when investigating Conceptual Expression (verbal), while the difference in Emotional Expression (non‐verbal) was not statistically significant. Here, the mean scores were also lower in the APD group than in the BPD group. The actual difference in expression between the two groups might be relatively small, requiring larger samples to detect it with certainty. Another explanation may be of a methodological nature, with the AII not being sensitive enough to capture the differences (e.g., in the operationalization of Expression on the AII, the score is reported as one opposed to the ACI, where the capacity to express oneself is operationalized into two communicational aspects). Therefore, it would be highly relevant to address this issue in future studies. Additionally, it has been established that the ACI and the AII tap into different dimensions of the AI construct (Frederiksen *et al*., 2021a); hence, further knowledge of the conceptual discrepancies between the ACI and the AII is needed.

While addressing levels of AI for discrete affects, we found that patients with APD scored significantly lower on Interest than those with BPD. This result was in line with findings from Johansen *et al*. (2013). When examining the *Access to Interest* scale, it was clear that the group with APD had significantly poorer access to this state than the BPD group. Considering the implications of a low capacity to process interest and to access its adaptive and motivational properties, we note that high levels of AI for Interest will motivate and guide the individual towards exploration, learning, and developing new skills (Izard, 1991). Additionally, the feeling of interest likely taps into the same construct domain as “the seeking system,” an affective organization much researched in animal models, which has also been associated with creativity (Panksepp & Watt, 2011; Reuter *et al*., 2005). According to Winnicott (1971), creativity is an essential ingredient in psychotherapeutic development. Accordingly, it is only when being creative that the patient can discover the true self. From this perspective, the low levels of Interest might be considered of central importance in understanding of and psychotherapeutic treatment for APD. Furthermore, while studying primary emotions in PDs, Karterud *et al*. (2016) identified a negative association between APD and Play and Seek. The lack of playfulness and seeking in APD was related to the high occurrence of Fear, suggesting that when fear dominates the mental landscape, it will inhibit play and seeking (Karterud *et al*., 2016).

Finally, regarding the *Access to Anger* scale, scores were lower in the APD group, although the difference was not statistically significant (*p*‐value = 0.055). It might still be of relevance to consider the implications and target this lack of access to anger in the psychotherapeutic treatment of APD. Reduced access to anger implies poor access to the motivational underpinnings of boundary formation and self‐assertion. When access to anger is poor, the experience of being angry typically ends in feelings of abandonment, resignation, anxiety or guilt (Solbakken, 2013).

### Emotional dysfunction in patients with BPD

We hypothesized that patients with BPD would primarily demonstrate emotional dysfunction in terms of being overwhelmed, paralyzed, and/or acting out. Our results showed that those with BPD had a significantly higher tendency to be driven by both jealousy and anger; in other words, our study supports the diagnostic notion of BPD causing an impaired ability to withhold aggressive impulses (American Psychiatric Association, 2013). Additionally, our results suggest that BPD is substantially more driven by feelings of interest than APD. Typically, this entails that states of interest or excitement led to doing things one later regrets or disregarding the needs and feelings of others because of one’s feelings of interest. In other words, this mode of experiencing interest also fits well with the notion of high and sometimes destructive impulsivity in BPD. On the other hand, one has to consider whether this finding is the expression of an actual incapacity in BPD to downregulate interest or whether it is more a question of its comparison to patients with APD, who knowingly struggle with adaptive management of Interest. Thus, in further studies, it is recommended to examine affective dysfunction in BPD in relation to other samples.

### Comparing emotional dysfunction in patients with APD or BPD

As a primary hypothesis of this study, we expected to find comparable overall levels of emotional dysfunction in the two groups of patients with APD and BPD, and the results support this hypothesis. On the other hand, we expected to identify differences between the groups in particular affects and prototypical modes of experiencing. Here, our hypotheses were confirmed, and it was evident that the most striking differences in emotional dysfunction between APD and BPD are due to variations in prototypical modes or patterns of experiencing and relating to one´s affects.

Our results are consistent with Simonsen *et al*. (2020), Sharp *et al*. (2015) and Wright, Hopwood, Skodol and Morey (2016) indicating the contribution of both general and more specific factors in personality pathology. In our view, emotional dysfunction as operationalized in the present study may effectively account for both general (e.g., Global AI) and specific aspects (e.g., Access to Interest) of BPD and APD. We also believe that our findings are particularly relevant to the upcoming ICD‐11 classification of PD severity (e.g., Bach & First, 2018; World Health Organization, 2018), which specifically relies on “Range and appropriateness of emotional experience and expression”, “Tendency to be emotionally over‐ or underreactive”, and “Ability to recognize and acknowledge unwanted emotions (e.g., anger, sadness).” The concept of AI appears to operationalize and assess all of these aspects of emotional dysfunction in a reliable and valid way (Frederiksen *et al*., 2021a, 2021b).

## STRENGTHS AND LIMITATIONS

Using the AII, the present study produced new evidence expanding and nuancing the existing understanding of emotional dysfunction in patients with APD or BPD. In particular, the identification of prototypical modes of experiencing, as measured by the *Access to* and *Driven by* scales, appears to enrich the empirical examination of emotional dysfunction in PDs. The present study is the first to make this distinction and systematically test it. However, prototypical modes of experiencing are only assessed for some of the affects represented in the AII. It would thus be of great value if additional scales assessing prototypical modes of experiencing all affects in the AII were developed.

A limitation of the present study is the relatively small sample size which increases the risk of type II errors. A larger sample might have allowed us to detect a difference between APD and BPD in regard to Expression and *Access to Anger*. Nevertheless, most of the theoretically meaningful differences were shown to be significantly different between the groups. Also, to increase the generalizability of the present findings, future studies should compare emotional dysfunction in APD and BPD to other clinical and nonclinical populations.

## CONCLUSIONS

The purpose of this study was to compare emotional dysfunction in patients with either APD or BPD. In line with our expectations, the results demonstrated that the two groups had similar overall levels of emotional dysfunction/AI. Furthermore, we expected that APD would report more dysfunction in the capacity to express and clearly communicate affective states; however, this difference was not found to be statistically significant. With respect to the level of discrete affects, it appeared that patients with APD had significantly more problems with the integration of Interest, while patients with BPD had significantly more problems with the integration of Jealousy. In terms of prototypical modes of experiencing affects, APD was characterized by a statistically significant lower access to Interest, while BPD was characterized by being more driven by Interest, Anger, and Jealousy. In conclusion, while APD and BPD are characterized by similar overall levels of emotional dysfunction, they differ systematically and predictably with respect to specific affects and modes of experiencing. Use of the AII provided evidence that expands and nuances our understanding of emotional dysfunction in patients with APD or BPD. Our findings also highlight specific areas of impairment that may be highly useful intervention targets for the treatment of APD and BPD.

We would like to thank psychologist Kenni Graversen for his efforts, advice and guidance during the design and data collection process. Also, we thank the staff at the Outpatient Clinic for Anxiety and Personality Disorders, Psychiatric Clinic North, for their positive attitude in implementing the study. Finally, we would like to thank the Outpatient Clinic for Personality Disorders, Psychiatric Clinic South and daily manager Brian Petersen for his contribution in the process. We want to thank Birgitte Christiansen, Maria Rodrigo Domingo and Martin Kamp Dalgaard for their contributions to this article.

The data that support the findings of this study are available from the corresponding author upon reasonable request.

## References

[sjop12771-bib-0001] American Psychiatric Association . (2013). Diagnostic and statistical manual of mental disorders (5th edn). Washington, DC: American Psychiatric Association.

[sjop12771-bib-0002] Bach, B. & First, M.B. (2018). Application of the ICD‐11 classification of personality disorders. BMC Psychiatry, 18, 351. 10.1186/s12888-018-1908-3 30373564 PMC6206910

[sjop12771-bib-0003] Bach, M. , de Zwaan, M. , Ackard, D. , Nutzinger, D.O. & Mitchell, J.E. (1994). Alexithymia: Relationship to personality disorders. Comprehensive Psychiatry, 35, 239–243. 8045115 10.1016/0010-440x(94)90197-x

[sjop12771-bib-0004] Baer, R.A. , Peters, J.R. , Eisenlohr‐Moul, T.A. , Geiger, P.J. & Sauer, S.E. (2012). Emotion‐related cognitive processes in borderline personality disorder: A review of the empirical literature. Clinical Psychology Review, 32, 359–369. 22561966 10.1016/j.cpr.2012.03.002

[sjop12771-bib-0005] Bagby, R.M. , Parker, J.D.A. & Taylor, G.J. (1994). The twenty‐item Toronto Alexithymia scale—I. Item selection and cross‐validation of the factor structure. Journal of Psychosomatic Research, 38, 23–32. 8126686 10.1016/0022-3999(94)90005-1

[sjop12771-bib-0006] Beck, A.T. & Freeman, A.M. (1990). Cognitive therapy of personality disorders. New York: Guilford Press.

[sjop12771-bib-0007] Berking, M. & Wupperman, P. (2012). Emotion regulation and mental health. Current Opinion in Psychiatry, 25, 128–134. 22262030 10.1097/YCO.0b013e3283503669

[sjop12771-bib-0008] Carpenter, R.W. & Trull, T.J. (2013). Components of emotion dysregulation in borderline personality disorder: a review. Current Psychiatry Reports, 15, 335. 10.1007/s11920-012-0335-2 23250816 PMC3973423

[sjop12771-bib-0009] Chapman, A.L. (2019). Borderline personality disorder and emotion dysregulation. Development and Psychopathology, 31, 1143–1156. 31169118 10.1017/S0954579419000658

[sjop12771-bib-0010] Chapman, A.L. , Rosenthal, M.Z. , Dixon‐Gordon, K.L. , Turner, B.J. & Kuppens, P. (2017). Borderline personality disorder and the effects of instructed emotional avoidance or acceptance in daily life. Journal of Personality Disorders, 31, 483–502. 27617652 10.1521/pedi_2016_30_264

[sjop12771-bib-0011] Cohen, J. (1988). Statistical power analysis for the behavioral sciences (2nd edn) . Hillsdale, NJ: Erlbaum Associates.

[sjop12771-bib-0012] Daros, A.R. & Williams, G.E. (2019). A meta‐analysis and systematic review of emotion‐regulation strategies in borderline personality disorder. Harvard Review of Psychiatry, 27, 217–232. 31219881 10.1097/HRP.0000000000000212

[sjop12771-bib-0013] Derogatis, L.R. (1994). SCL‐90‐R: Administration, scoring and procedures manual (3rd edn). Minneapolis, MN: NCS Pearson.

[sjop12771-bib-0014] Dimaggio, G. , Procacci, M. , Nicolò, G. , Popolo, R. , Semerari, A. , Carcione, A. *et al* (2007). Poor metacognition in narcissistic and avoidant personality disorders: Four psychotherapy patients analysed using the Metacognition Assessment Scale. Clinical Psychology & Psychotherapy, 14, 386–401.

[sjop12771-bib-0015] First, M.B. (1994). Structured and clinical interview for DSM‐IV Axis II personality disorders (version 2.0.). New York: New York State Psychiatry Institute.

[sjop12771-bib-0016] Frederiksen, C. Solbakken, O.A., Licht, R.W. , Christensen, A.-E. , Jørgensen, C.R. , & Kjaersdam, G.T. (2001a) Validation of the Affect Integration Inventory in a sample of patients with personality disorders: a cross sectional study, in press. 10.1016/j.actpsy.2022.103554 35276544

[sjop12771-bib-0017] Frederiksen, C. , Solbakken, O.A. , Licht, R.W. , Jørgensen, C.R. , Rodrigo‐Domingo, M. & Kjaersdam Telléus, G. (2021b). The relationship between affect integration and psychopathology in patients with personality disorder: A cross‐sectional study. Medicina, 57, 627. 10.3390/medicina57060627 PMC8234290 34208658

[sjop12771-bib-0018] Greenberg, L.S. & Bolger, E. (2001). An emotion‐focused approach to the overregulation of emotion and emotional pain. Journal of Clinical Psychology, 57, 197–211. 11180147 10.1002/1097-4679(200102)57:2<197::aid-jclp6>3.0.co;2-o

[sjop12771-bib-0019] Herpertz, S.C. , Schwenger, U.B. , Kunert, H.J. , Lukas, G. , Gretzer, U. , Nutzmann, J. *et al* (2000). Emotional responses in patients with borderline as compared with avoidant personality disorder. Journal of Personality Disorders, 14, 339–351. 11204341 10.1521/pedi.2000.14.4.339

[sjop12771-bib-0020] Horowitz, L.M. , Alden, L.E. , Wiggins, J.S. & Pincus, A.L. (2000). Inventory of interpersonal problems manual. Odessa, FL: The Psychological Corporation.

[sjop12771-bib-0021] Izard, C.E. (1991). The psychology of emotions. New York: Plenum Press.

[sjop12771-bib-0022] Johansen, M.S. , Normann‐Eide, E. , Normann‐Eide, T. & Wilberg, T. (2013). Emotional dysfunction in avoidant compared to borderline personality disorder: a study of affect consciousness. Scandinavian Journal of Psychology, 54, 515–521. 24107113 10.1111/sjop.12076

[sjop12771-bib-0023] Jordet, H. & Ladegård, P. (2018). En Mentaliseringsbasert Forståelse av unnvikende personlighetsforstyrrelse. Matrix, 34, 4–19.

[sjop12771-bib-0024] Joyce, A.S. , Fujiwara, E. , Cristall, M. , Ruddy, C. & Ogrodniczuk, J.S. (2013). Clinical correlates of alexithymia among patients with personality disorder. Psychotherapy Research, 23, 690–704. 23731378 10.1080/10503307.2013.803628

[sjop12771-bib-0025] Karterud, S. , Pedersen, G. , Johansen, M. , Wilberg, T. , Davis, K. & Panksepp, J. (2016). Primary emotional traits in patients with personality disorders. Personality and Mental Health, 10, 261–273. 27257161 10.1002/pmh.1345

[sjop12771-bib-0026] Lampe, L. & Malhi, G.S. (2018). Avoidant personality disorder: Current insights. In Psychology research and behavior management (vol 11, pp. 55–56). Dove Medical Press Ltd. 10.2147/PRBM.S121073 29563846 PMC5848673

[sjop12771-bib-0027] Lech, B. , Andersson, G. & Holmqvist, R. (2008). Consciousness about own and others’ affects: A study of the validity of a revised version of the Affect Consciousness Interview. Scandinavian Journal of Psychology, 49, 515–521. 18489533 10.1111/j.1467-9450.2008.00666.x

[sjop12771-bib-0028] Linehan, M.M. (1993). Cognitive‐behavioral treatment of borderline personality disorder. New York: Guilford Press.

[sjop12771-bib-0029] Links, P.S. , Gould, B. & Ratnayake, R. (2003). Assessing suicidal youth with antisocial, borderline, or narcissistic personality disorder. Canadian Journal of Psychiatry. Revue Canadienne de Psychiatrie, 48, 301–310. 12866335 10.1177/070674370304800505

[sjop12771-bib-0030] Millon, T. (1981). Disorders of personality: DSM‐III, Axis II. New York: Wiley‐Interscience. https://books.google.dk/books?id=W3hHAAAAMAAJ

[sjop12771-bib-0031] Monsen, J.T. & Monsen, K. (1999). Affects and affect consciousness: A psychotherapy model integrating Silvan Tomkins’ affect‐ and script theory witin the framework of self psychology. In A. Goldberg (Ed.), Pluralism in self psychology: Progress in self psychology (vol 15, pp. 287–307). Hillsdale, NJ: Analytic Press.

[sjop12771-bib-0032] Monsen, J.T. , Eilertsen, D.E. , Melgård, T. & Ødegård, P. (1996). Affects and affect consciousness: Initial experiences with the assessment of affect integration. The Journal of Psychotherapy Practice and Research, 5, 238–249. 22700292 PMC3330421

[sjop12771-bib-0033] Moroni, F. , Procacci, M. , Pellecchia, G. , Semerari, A. , Nicolò, G. , Carcione, A. *et al* (2016). Mindreading dysfunction in avoidant personality disorder compared with other personality disorders. The Journal of Nervous and Mental Disease, 204, 752–757. 27227557 10.1097/NMD.0000000000000536

[sjop12771-bib-0034] Nicolo, G. , Semerari, A. , Lysaker, P.H. , Dimaggio, G. , Conti, L. , D’Angerio, S. *et al* (2011). Alexithymia in personality disorders: correlations with symptoms and interpersonal functioning. Psychiatry Research, 190, 37–42. 20800288 10.1016/j.psychres.2010.07.046

[sjop12771-bib-0035] Nørgaard, N.L. & Simonsen, S. (2019). Den ængstelige personlighed. Copenhagen, Denmark: Hans Reitzels Forlag.

[sjop12771-bib-0036] Panksepp, J. (2005). Affective consciousness: Core emotional feelings in animals and humans. Consciousness and Cognition, 14, 30–80. 15766890 10.1016/j.concog.2004.10.004

[sjop12771-bib-0037] Panksepp, J. & Watt, D. (2011). What is basic about basic emotions? Lasting lessons from affective neuroscience. Emotion Review, 3, 387–396.

[sjop12771-bib-0038] Procacci, M. , Popolo, R. , Petrilli, D. & Dimaggio, G. (2007). Avoidant personality disorder: model and treatment. In G. Dimaggio , A. Semerari , A. Carcione , G. Nicolò & M. Procacci (Eds.), Psychotherapy of personality disorders. England, UK: Routledge.

[sjop12771-bib-0039] Reuter, M. , Panksepp, J. , Schnabel, N. , Kellerhoff, N. , Kempel, P. & Hennig, J. (2005). Personality and biological markers of creativity. European Journal of Personality, 19, 83–95.

[sjop12771-bib-0040] Rosenthal, M.Z. , Cukrowicz, K.C. , Cheavens, J.S. & Lynch, T.R. (2006). Self‐punishment as a regulation strategy in borderline personality disorder. Journal of Personality Disorders, 20, 232–246. 16776553 10.1521/pedi.2006.20.3.232

[sjop12771-bib-0041] Sansone, R.A. & Sansone, L.A. (2011). Substance use disorders and borderline personality: common bedfellows. Innovations in Clinical Neuroscience, 8, 10–13. PMC3196330 22010059

[sjop12771-bib-0042] SCAN Advisory Committee . (2002). Present state examination: Kort version til klinisk brug (3rd edn). Brøndby, Denmark: Glaxo Smith Kline.

[sjop12771-bib-0043] Schoenleber, M. & Berenbaum, H. (2010). Shame aversion and shame‐proneness in Cluster C personality disorders. Journal of Abnormal Psychology, 119, 197–205. 20141256 10.1037/a0017982

[sjop12771-bib-0044] Schoenleber, M. & Berenbaum, H. (2012). Aversion and proneness to shame in self‐ and informant‐reported personality disorder symptoms. Personality Disorders: Theory, Research, and Treatment, 3, 294–304. 10.1037/a0025654 22452760

[sjop12771-bib-0045] Sharp, C. , Wright, A.G.C. , Fowler, J.C. , Frueh, B.C. , Allen, J.G. , Oldham, J. *et al* . (2015). The structure of personality pathology: Both general (‘g’) and specific (‘s’) factors? Journal of Abnormal Psychology, 124, 387–398. 25730515 10.1037/abn0000033

[sjop12771-bib-0046] Simonsen, S. , Eikenaes, I.‐ U.‐M. , Bach, B. , Kvarstein, E. , Gondan, M. , Møller, S.B. *et al* (2020). Level of alexithymia as a measure of personality dysfunction in avoidant personality disorder. Nordic Journal of Psychiatry, 75, 266–274. 33146059 10.1080/08039488.2020.1841290

[sjop12771-bib-0047] Solbakken, O.A. (2013). Arbeid med følelser–integrerende element i psykoterapi. In I.K. Benum , E. Dalsgaard Axelsen & E. Hartmann (Eds.), God psykoterapi: et integrativt perspektiv (pp. 142–171). Oslo, Norway: Pax.

[sjop12771-bib-0048] Solbakken, O.A. , Hansen, R.S. & Monsen, J.T. (2011). Affect integration and reflective function: Clarification of central conceptual issues. Psychotherapy Research, 21, 482–496. 21623546 10.1080/10503307.2011.583696

[sjop12771-bib-0049] Solbakken, O.A. , Hansen, R.S. , Havik, O.E. & Monsen, J.T. (2011). Assessment of affect integration: Validation of the affect consciousness construct. Journal of Personality Assessment, 93, 257–265. 21516584 10.1080/00223891.2011.558874

[sjop12771-bib-0050] Solbakken, O.A. & Monsen, J. (2021). Validation of a short form of the Affect Integration Inventory. International Journal of Psychology & Psychological Therapy, 21, 107–122.

[sjop12771-bib-0051] Solbakken, O.A. , Rauk, M. , Solem, A. , Lødrup, W. & Monsen, J.T. (2017). Tell me how you feel and I will tell you who you are : First validation of the affect integration inventory. Scandinavian Psychologist, 4, 1–31.

[sjop12771-bib-0052] Spinhoven, P. , Bamelis, L. , Molendijk, M. , Haringsma, R. & Arntz, A. (2009). Reduced specificity of autobiographical memory in Cluster C personality disorders and the role of depression, worry, and experiential avoidance. Journal of Abnormal Psychology, 118, 520–530. 19685949 10.1037/a0016393

[sjop12771-bib-0053] StataCorp . (2019). Stata statistical software: Release 16. College Station, TX: StataCorp.

[sjop12771-bib-0054] Taarvig, E. , Solbakken, O.A. , Grova, B. & Monsen, J.T. (2015). Affect Consciousness in children with internalizing problems: Assessment of affect integration. Clinical Child Psychology and Psychiatry, 20, 591–610. 24941941 10.1177/1359104514538434

[sjop12771-bib-0055] Taylor, C.T. , Laposa, J.M. & Alden, L.E. (2004). Is avoidant personality disorder more than just social avoidance? Journal of Personality Disorders, 18, 571–594. 15615668 10.1521/pedi.18.6.571.54792

[sjop12771-bib-0056] Tomkins, S.S. (2008a). Affect imagery consciousness: The complete edition (vols I and II). Springer Publishing.

[sjop12771-bib-0057] Tomkins, S.S. (2008b). Affect imagery consciousness: The complete edition (vols III and IV). Springer Publishing.

[sjop12771-bib-0058] Wilberg, T. , Karterud, S. , Pedersen, G. & Urnes, Ø. (2009). The impact of avoidant personality disorder on psychosocial impairment is substantial. Nordic Journal of Psychiatry, 63, 390–396. 19333817 10.1080/08039480902831322

[sjop12771-bib-0059] Winnicott, D.W. (1971). Playing and reality. Basic Books, New York: Penguin.

[sjop12771-bib-0060] World Health Organization (2018). International classification of diseases for mortality and morbidity statistics (11th Revision). Retrieved from https://icd.who.int/browse11/l‐m/en. Accessed April 2, 2019.

[sjop12771-bib-0061] World Health Organization, Division of Mental Health (1996). WHOQOL‐BREF : introduction, administration, scoring and generic version of the assessment : field trial version, December 1996. World Health Organization. https://apps.who.int/iris/handle/10665/63529. Accessed April 2, 2019.

[sjop12771-bib-0062] Wright, A.G.C. , Hopwood, C.J. , Skodol, A.E. & Morey, L.C. (2016). Longitudinal validation of general and specific structural features of personality pathology. Journal of Abnormal Psychology, 125, 1120–1134. 27819472 10.1037/abn0000165 PMC5119768

[sjop12771-bib-0063] Ye, G. , Yao, F. , Wen‐Qing, F. & Kong, M. (2011). The relationships of self‐esteem and affect of university students with avoidant personality disorder. Chinese Mental Health Journal, 25, 141–145.

